# Skin wound healing as a mirror to cardiac wound healing

**DOI:** 10.1113/EP090888

**Published:** 2023-04-24

**Authors:** Merry L. Lindsey, Mediha Becirovic‐Agic

**Affiliations:** ^1^ School of Graduate Studies Meharry Medical College Nashville Tennessee USA; ^2^ Research Service Nashville VA Medical Center Nashville Tennessee USA; ^3^ Integrative Physiology, Department of Medical Cell Biology Uppsala University Uppsala Sweden

**Keywords:** dermatology, heart, heart failure, myocardial infarction, physiology, proteomics

## Abstract

Wound healing is a general response of the body to injury. All organs share in common three response elements to wound healing: inflammation to prevent infection and stimulate the removal of dead cells, active anti‐inflammatory signalling to turn off the inflammatory response, and a repair phase characterized by extracellular matrix scar formation. The extent of scar formed depends on the ability of endogenous cells that populate each organ to regenerate. The skin has keratinocytes that have regenerative capacity, and in general, wounds are fully re‐epithelialized. Heart, in contrast, has cardiac myocytes that have little to no regenerative capacity, and necrotic myocytes are entirely replaced by scars. Despite differences in tissue regeneration, the skin and heart share many wound‐healing properties that can be exploited to predict the cardiac response to pathology. We summarize in this review article our current understanding of how the response of the skin to a wounding event can inform us about the ability of the myocardium to respond to a myocardial infarction.

## Abstract (highlights)

- **What is the topic of this review?**
  Wound healing is a general response of the body to injury and can be divided into three phases: inflammation, inflammation resolution and repair. In this review, we compare the wound‐healing response of the skin after an injury and the wound‐healing response of the heart after a myocardial infarction.
- **What advances does it highlight?**
  We highlight differences and similarities between skin and cardiac wound healing and summarize how skin can be used to provide information about the heart.

## 1 INTRODUCTION

All tissues in the body respond to injury by activating a general wound‐healing response. The wound‐healing response includes inflammation, inflammation resolution and tissue repair (Czubryt, 2012; Richardson, 2018). Inflammation is characterized by immune cell infiltration necessary for preventing infection. Inflammation also serves to bring in proteases that remove damaged cells and debris to make room for new tissue (Chalise et al., 2023; Schreml et al., 2010). The resolution of inflammation is characterized by the active secretion of anti‐inflammatory molecules that turn off the inflammatory response and thereby initiate tissue repair (Chalise et al., 2023; Schreml et al., 2010). During tissue repair, new extracellular matrix (ECM) is formed, and tissue is regenerated to the extent possible for that particular tissue (Chalise et al., 2023; Schreml et al., 2010). Within the general wound‐healing response, there is an organ‐specific continuum of outcomes, ranging from full tissue regeneration at one end of the spectrum to very limited regeneration and scar replacement at the other end.

Full tissue regeneration occurs when there is a complete replacement of the damaged tissue by original endogenous cell types, resulting in no trace of injury. On this side of the wound‐healing spectrum is the skin, along with the liver, intestines and skeletal muscle (Baddour et al., 2012; Poss, 2010). These organs are able to regenerate due to progenitor cells within the tissue that proliferate and differentiate into the cells that populate the organ (Baddour et al., 2012; Poss, 2010). The skin, for example, contains epidermal progenitor cells that undergo cyclical proliferation and differentiate into keratinocytes, a process that is essential to maintain the barrier function of the skin. Upon injury, the progenitor cells can quickly proliferate, migrate and differentiate to regenerate the epidermis. Consequently, small and superficial skin wounds affecting only the epidermis can be restored without any scar formation. Deeper wounds that involve the dermis are not capable of full regeneration and are repaired with some scar formation (Chen et al., 2009).

The intestines contain stem cells that proliferate rapidly after injury and differentiate into all cell types necessary for rebuilding the intestinal wall (Baddour et al., 2012). Likewise, skeletal muscle contains satellite cells that are activated upon injury, differentiate into myoblasts and fuse with existing myofibres to replace damaged muscle (Poss, 2010). The liver differs in its regenerative process, in that it regenerates after injury through the proliferation of remaining hepatocytes and not by a specialized stem cell population (Baddour et al., 2012).

The heart, brain and kidneys are on the other side of the wound‐healing regenerative capacity spectrum (Baddour et al., 2012). The heart is, for the most part, not capable of regeneration, because cardiomyocytes are terminally differentiated cells with limited ability to re‐enter mitosis and restore the damaged tissue by proliferation. Consequently, necrotic cardiomyocytes are replaced with connective tissue scar, and surviving cardiomyocytes compensate by increasing in size by undergoing hypertrophy (Baddour et al., 2012; Poss, 2010).

The mammalian brain does exhibit neurogenesis in some regions, including the hippocampus and subventricular zone. The subventricular zone also contains pluripotent stem cells that can give rise to new neurons, astrocytes and oligodendrocytes (Zamproni et al., 2021). Despite these capabilities, the regenerative capacity of the brain in response to injury is very limited. This is attributable to the formation of the glial scar, which is necessary to prevent further expansion of neuronal damage but, at the same time, provides a hostile environment for axonal growth and remyelination and for stem cell integration, thus inhibiting regeneration (Zamproni et al., 2021). Owing to the plasticity of the brain, the surviving neurons compensate for damage by undergoing both molecular and synaptic changes in order to recreate functional neuronal circuits (Baddour et al., 2012; Zamproni et al., 2021).

Despite differences in regenerative capacity in different organs, dysregulation of the general wound‐healing response results in similar outcomes in all organs. For optimal tissue repair, injured cells need to be removed in a timely manner with some but not excessive inflammation (Chalise et al., 2023; Czubryt, 2012; Martin, 1997). Pro‐inflammatory cells need to undergo apoptosis, and there needs to be a switch to an anti‐inflammatory/reparative phenotype to resolve inflammation in a timely manner, which, in turn, stimulates fibroblasts to form new ECM and endothelial cells to revascularize the tissue (Chalise et al., 2023; Czubryt, 2012; Martin, 1997). Prolonged inflammation, delayed inflammation resolution or impaired angiogenesis can each prevent or limit repair. Insufficient ECM deposition and crosslinking results in a weak scar, whereas excessive ECM deposition and crosslinking results in fibrosis and a stiff scar (Chalise et al., 2023; Czubryt, 2012; Martin, 1997; Prabhu & Frangogiannis, 2016).

**FIGURE 1:**
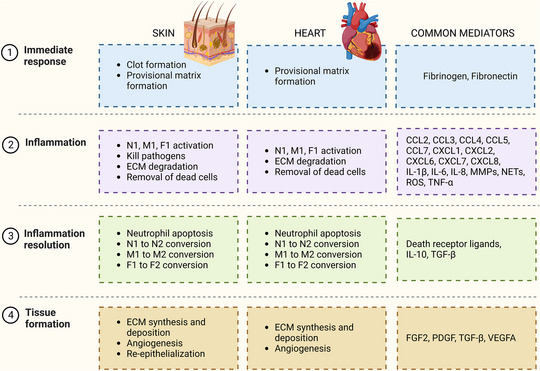
Summary of the wound‐healing processes after a skin injury and after myocardial infarction, highlighting common mediators. Abbreviations: CCL, C‐C motif chemokine ligand; CXCL, C‐X‐C motif chemokine ligand; ECM, extracellular matrix; FGF, fibroblast growth factor; F1, pro‐inflammatory fibroblasts; F2, anti‐inflammatory/reparative fibroblasts; IL, interleukin; MMP, matrix metalloproteinase; M1, pro‐inflammatory macrophages; M2, anti‐inflammatory/reparative macrophages; NETs, neutrophil extracellular traps; N1, pro‐inflammatory neutrophils; N2, anti‐inflammatory/reparative neutrophils; PDGF, platelet‐derived growth factor; ROS, reactive oxygen species; TGF, transforming growth factor; TNF, tumour necrosis factor; VEGFA, vascular endothelial growth factor A.

Although the skin and the heart are at opposite ends of the regeneration spectrum, the similarities in their wound‐healing responses provide an opportunity to explore the concept that how the skin responds to an injury can serve as a gauge to predict the cardiac response to wounding. Furthermore, diabetes is a predictor of the future development of heart failure after myocardial infarction (MI) in humans, and patients with diabetes have impaired skin wound healing (Lewis et al., 2003). We recently published an evaluation showing that mice exhibiting faster skin wound healing also had better survival and repair after MI (Becirovic‐Agic et al., 2022). Myocardial infarction occurs when a coronary artery is occluded for sufficient time to cause hypoxia and induce cardiomyocyte necrosis. The occlusion results from atherosclerotic plaque build‐up or rupture of an atherosclerotic plaque forming a thrombus and occluding a distal coronary artery (Montecucco et al., 2016). Insight into the wound‐healing mechanism of the heart can be obtained by examining the wound‐healing mechanism of the skin. In this review, we compare skin and MI cardiac wound healing, highlighting differences and similarities and summarizing how skin can be used to provide information about the heart (Figure 1).

## 2 CELLULAR COMPOSITION OF THE SKIN AND THE HEART

Normal skin is primarily composed of ECM, which provides unique properties of elasticity, tensile strength and compressibility. The skin is made of three layers: the epidermis, dermis and hypodermis (Chen et al., 2009; Martin, 1997). The epidermis is the top layer and consists of several layers of epithelial cells, which extend from the basement membrane localized between the epidermis and dermis. Within the basement membrane, there are progenitor cells that undergo continuous proliferation and differentiation into keratinocytes for normal turnover of skin. As keratinocytes migrate towards the surface, they undergo differentiation, maturation and keratinization (Chen et al., 2009). The dermis, located below the epidermis, is a connective tissue layer and consists of dermal fibroblasts, ECM, blood vessels and skin appendages, such as hair follicles and sebaceous or sweat glands (Chen et al., 2009). The hypodermis is located below the dermis and consists mainly of adipose tissue to provide insulation and cushioning between the skin and skeletal structures (Chen et al., 2009).

The heart is composed of about one‐third cardiomyocytes by cell number (depending on species) and ∼90% by volume. The remaining two‐thirds by cell number are fibroblasts, smooth muscle cells, endothelial cells, neurons and immune cells (O'Rourke et al., 2019). In normal conditions, the primary ECM in the heart is basement membrane surrounding the cells and perivascular ECM surrounding blood vessels (Lindsey et al., 2018; Silva et al., 2021). Cardiomyocytes are responsible for the contractile force of the heart, and after ∼1 week after birth are set in number for the lifespan of the organism (Parmacek & Epstein, 2009). Their proliferative capacity is minimal, which is the reason for the low regenerative capacity of the heart (Baddour et al., 2012).

## 3 WOUND HEALING PART 1: IMMEDIATE RESPONSE TO INJURY (MINUTES TO HOURS AFTER INJURY)

The immediate response to a skin injury is platelet activation, coagulation and clot formation, which limits blood loss and invasion of pathogens (Czubryt, 2012; Martin, 1997). The clot, rich in platelets, is stabilized by a mesh of crosslinked fibrin fibres with smaller amounts of fibronectin, vitronectin and thrombospondin (Martin, 1997; Rodero & Khosrotehrani, 2010). These proteins form a provisional matrix that serves as a scaffold for infiltrating cells, allowing migration during the repair process (Martin, 1997). The clot also serves as a reservoir for cytokines, chemokines and growth factors [platelet‐derived growth factor (PDGF), vascular endothelial growth factor (VEGF), insulin‐like growth factor‐1 (IGF‐1) and transforming growth factor β (TGF‐β)], which are released by activated platelets (Czubryt, 2012; Kondo & Ishida, 2010; Martin, 1997). This cocktail initiates the repair process by stimulating immune cell infiltration, re‐epithelization and connective tissue contraction (Gushiken et al., 2021; Kondo & Ishida, 2010; Martin, 1997). In addition to platelets, damaged keratinocytes also stimulate immune cell infiltration by releasing damage‐associated molecular patterns (DAMPs), high‐mobility group box 1 protein, interleukin (IL)‐1β, IL‐6 and tumour necrosis factor‐α (TNF‐α) (Gushiken et al., 2021; Kondo & Ishida, 2010).

Myocardial infarction occurs owing to coronary artery occlusion and subsequent hypoxia, yielding cardiomyocyte necrosis when the hypoxia is of sufficient duration. Although there is no major bleeding, platelets accumulate in the infarct region, starting at 6 h and peaking at 72 h in a permanent coronary artery ligation mouse model (Liu et al., 2011). The contribution of platelets to MI wound healing is an emerging field, with platelet subtypes taking different roles (Liu et al., 2011; Schanze et al., 2022). During MI, the coagulation cascade is activated by endothelial damage, resulting in extravasation and accumulation of plasma proteins, such as fibrinogen and fibronectin (Richardson, 2018). These proteins form a provisional matrix that serves as a scaffold and allows migration of infiltrating and proliferating cells (Prabhu & Frangogiannis, 2016), similar to the provisional matrix formed within the clot during skin wound healing. After MI, DAMPs from necrotic cardiomyocytes are the primary stimulator of immune cell infiltration to initiate wound healing (Becirovic‐Agic et al., 2021; Chalise et al., 2023).

## 4 WOUND HEALING PART 2: INFLAMMATION

The necrotic cells need to be removed to make room for new scar or regenerated tissue, and inflammation is essential for this to occur. The inflammatory response is initiated within a few hours of tissue injury and is very similar after a skin injury or MI. Immune cells infiltrate the injured tissue and release chemokines and cytokines that create a pro‐inflammatory microenvironment, in addition to proteases that enzymatically break down ECM surrounding the necrotic cells. Proteases also serve to fine‐tune inflammation by activating and inactivating cytokines and chemokines (Chalise et al., 2023; Gillitzer & Goebeler, 2001; Gushiken et al., 2021). A pro‐inflammatory microenvironment promotes ECM degradation and tissue debridement and is therefore required to make room for new tissue. An insufficient inflammatory response results in a weak scar, whereas an overactive inflammatory response can extend out to damage the surrounding healthy tissue, induce excess ECM degradation and prevent resolution of inflammation. Thus, a balanced inflammatory response is crucal for optimal skin and cardiac wound healing.

Neutrophils are the first immune cells to infiltrate both the skin wound and the MI. At day 1, neutrophils are the predominant cell type in the injury site (Chalise et al., 2023; Czubryt, 2012; Daseke et al., 2019; Gillitzer & Goebeler, 2001). During skin wound healing, degradation products from pathogens are strong attractants for neutrophils (Rodero & Khosrotehrani, 2010). Given that MI induces an intense sterile inflammation (Prabhu & Frangogiannis, 2016), degradation products from pathogens play little role in attracting neutrophils, while DMAPs released from necrotic myocytes play a bigger role (Richardson, 2018). C‐X‐C chemokine (CXC) receptor CXCR2 binding chemokines (CXCL1, CXCL2, CXCL6, CXCL7 and CXCL8) are crucial for recruitment of neutrophils for both skin wounds and MI (Gillitzer & Goebeler, 2001; Liehn et al., 2011; Prabhu & Frangogiannis, 2016). Extravasated neutrophils release reactive oxygen species (ROS) and form neutrophil extracellular traps (NETs) to kill and clear contaminating bacteria (Bonaventura et al., 2020; Czubryt, 2012; Martin, 1997). Although pathogens are not present after MI, activated neutrophils release ROS, and DAMPs stimulate NET formation (Bonaventura et al., 2020); both are important in MI cell communication. Neutrophils secrete proteases [e.g., matrix metalloproteinase (MMP)‐8, MMP‐9, myeloperoxidase and neutrophil elastase] that degrade ECM to fragments, thus facilitating removal of dead cells (Chalise et al., 2023; Daseke et al., 2019; Wilgus et al., 2013). Furthermore, neutrophils release pro‐inflammatory cytokines [IL‐1β, TNF‐α, IL‐8, C‐C motif chemokine ligand (CCL)3 and CCL5] to stimulate leucocyte infiltration further (Chalise et al., 2023; Gillitzer & Goebeler, 2001; Gushiken et al., 2021). Despite the importance of these functions for tissue repair, the explosion of inflammatory mediators released from neutrophils can exacerbate tissue damage (Leoni & Soehnlein, 2018; Liehn et al., 2011; Wilgus et al., 2013). Excessive neutrophil activation has been linked to prolonged inflammation and chronic skin wounds, and in the case of MI, to wall thinning and cardiac rupture (Chalise et al., 2021). To limit the negative effects of neutrophils, it is therefore necessary that neutrophils undergo timely apoptosis and convert to anti‐inflammatory phenotypes (Chalise et al., 2022, 2021; Ma et al., 2016).

Around day 3 after tissue injury, when neutrophils start to decrease, there is a massive infiltration of monocytes/macrophages (Chalise et al., 2023; Gillitzer & Goebeler, 2001). Recruitment of monocytes/macrophages is stimulated by CC chemokines (CCL2, CCL3, CCL4, CCL5 and CCL7) during both dermal injury and MI (Chen & Frangogiannis, 2021; Gillitzer & Goebeler, 2001; Kubota & Frangogiannis, 2022; Martin, 1997). Early macrophages release MMP‐8 and MMP‐9 to aid neutrophils in further degradation of ECM, phagocytosis of bacteria (for skin wounds) and removal of necrotic tissue (Chalise et al., 2023; Czubryt, 2012; Martin, 1997). The intense phagocytic activity of macrophages is linked to improved skin and cardiac wound healing, and reduced phagocytic capacity in macrophages is linked to chronic skin wounds and poor left ventricular remodelling (Jung et al., 2017; Kubota & Frangogiannis, 2022; Lindsey et al., 2019; Mouton et al., 2018; Rodero & Khosrotehrani, 2010). At the same time, early macrophages are significant promoters of pro‐inflammatory environment, because macrophages stimulated by pro‐inflammatory cytokines produce and release more pro‐inflammatory cytokines (Chalise et al., 2023; Gillitzer & Goebeler, 2001; Martin, 1997). Macrophages produce a number of pro‐inflammatory cytokines, including IL‐1β, IL‐6 and TNF‐α. After a dermal injury, these cytokines stimulate fibroblasts to produce keratinocyte growth factor to stimulate re‐epithelization (Rodero & Khosrotehrani, 2010). Later during the wound‐healing process, macrophages switch phenotype and become anti‐inflammatory/reparative, which is crucial for resolution of inflammation and initiation of scar formation (Mouton et al., 2018).

In addition to immune cells, cardiac fibroblasts also contribute to the pro‐inflammatory environment and tissue debridement. Ischaemia, DAMPs, pro‐inflammatory cytokines (IL‐1α, IL‐1β and TNF‐α) and ROS activate and induce a pro‐inflammatory phenotype in cardiac resident fibroblasts (Daseke et al., 2020; Ma et al., 2017; Mouton et al., 2019). Pro‐inflammatory fibroblasts release proteases that degrade ECM (Ma et al., 2017). Furthermore, the inflammatory microenvironment inhibits the conversion of pro‐inflammatory fibroblasts to reparative fibroblasts and inhibits ECM synthesis and deposition, thus indirectly promoting ECM degradation (Daseke et al., 2020; Mouton et al., 2019).

## 5 WOUND HEALING PART 3: INFLAMMATION RESOLUTION

An anti‐inflammatory microenvironment stimulates ECM synthesis and deposition, and thereby, tissue regeneration and scar formation. Therefore, resolution of inflammation is crucial for initiating tissue repair. An anti‐inflammatory environment is promoted by active secretion of anti‐inflammatory and pro‐resolving mediators necessary for polarizing inflammatory cells towards an anti‐inflammatory/reparative phenotype. This process is very similar during skin wound healing and MI repair.

Although neutrophils are important for initiating the wound‐healing response both after a skin injury and after MI, it is crucial that the pro‐inflammatory neutrophils undergo timely apoptosis for resolution of inflammation to occur. Neutrophils are evolutionarily designed to have a short lifespan and undergo spontaneous apoptosis to prevent prolonged inflammation (Daseke et al., 2021). During inflammatory conditions, the lifespan of neutrophils can be prolonged by exposure to pro‐survival stimuli, such as granulocyte‐colony stimulating factor (GCSF), granulocyte macrophage‐colony stimulating factor (GM‐CSF) and interferon‐γ (IFN‐γ) (Colotta et al., 1992). Neutrophil apoptosis can be induced by several factors, including death receptor ligands TNF‐α and Fas, and by MMP‐12 released by infiltrating macrophages (Chalise et al., 2022; Daseke et al., 2021).

Apoptotic neutrophils provide a strong signal for resolution of inflammation. Apoptotic neutrophils alter the microenvironment by expressing scavenging receptors that bind and deplete inflammatory molecules (Becirovic‐Agic et al., 2021). Furthermore, ingestion of apoptotic neutrophils drives pro‐inflammatory macrophages to adopt an anti‐inflammatory and reparative phenotype (Chalise et al., 2023). These macrophages reduce production of pro‐inflammatory IL‐1β and TNF‐α and, at the same time, increase production of anti‐inflammatory IL‐10 and TGF‐β (Hulsmans et al., 2016). The anti‐inflammatory and reparative macrophages are essential for initiating tissue formation. Anti‐inflammatory and reparative macrophages release platelet‐derived growth factor and vascular endothelial growth factor A (VEGFA) and thereby stimulate blood vessel formation (Chalise et al., 2023). Furthermore, macrophages stimulate activation of resident fibroblasts and their recruitment from the border zone by releasing CCL7 and CCL8 (O'Rourke et al., 2019). Anti‐inflammatory and reparative macrophages also release TGF‐β to stimulate the conversion of pro‐inflammatory fibroblasts into reparative fibroblasts (Becirovic‐Agic et al., 2021; Chalise et al., 2023).

## 6 WOUND HEALING PART 4: TISSUE REPAIR

The final step of the wound‐healing process is tissue repair. During this step, new ECM is synthesized, deposited and crosslinked; the scar is revascularized; and, in the case of skin, dermal epithelial cells are regenerated. In both skin and heart, wound healing leads to formation of a fibrotic collagen‐rich scar (Richardson, 2018). Differences in the two organs lie in the extent to which the damaged tissue is repopulated by cells or scar. Dermal wounds are, for the most part, entirely repopulated by keratinocytes, fibroblasts, endothelial cells and smooth muscle cells and can be restored to >80% of the pre‐injury state. The extent of regeneration is dependent on the depth of the wound and how the wound healing proceeds (Chen et al., 2009). Myocardial infarct scars are repopulated by fibroblasts, endothelial cells and smooth muscle cells, whereas repopulation by cardiomyocytes is negligible, resulting in a revascularized collagen‐rich scar that is physiologically suboptimal compared with the pre‐MI state, but crucial for preventing left ventricular aneurysm and cardiac rupture (Chalise et al., 2023). In the heart, a new homeostasis is established and maintained indefinitely.

### 6.1 Extracellular matrix synthesis and deposition

Within a week of skin injury, the fibrin clot is dissolved and replaced by fibroblasts (Martin, 1997). The fibroblasts are recruited by growth factors [e.g., PDGF, fibroblast growth factor (FGF) and TGF‐β] that stimulate fibroblasts to proliferate, convert to reparative fibroblasts and synthesize high concentrations of ECM (desJardins‐Park et al., 2018; Kondo & Ishida, 2010). Polarized fibroblasts have a significantly higher capacity to produce ECM components in comparison to regular fibroblasts (desJardins‐Park et al., 2018; Kondo & Ishida, 2010). In addition, polarized fibroblasts are able to contract owing to their expression of α‐smooth muscle actin, which brings wound edges together to reduce the area that needs to be closed by re‐epithelization (Chen et al., 2009). As fibroblasts accumulate, they produce collagen that gradually replaces the fibrin matrix (Chen et al., 2009; Czubryt, 2012). Early during the healing process, collagen type III predominates and is later replaced by collagen type I and elastin, which gives a mechanically stronger and more elastic scar (Gushiken et al., 2021). Over time, the collagen is crosslinked to strengthen the scar further (desJardins‐Park et al., 2018). Fibroblasts isolated from chronic skin wounds have lower proliferative capacity and undergo earlier senescence in comparison to fibroblasts isolated from acute skin wounds (Wall et al., 2008). Fibroblasts isolated from hypertrophic wounds, on the contrary, have increased proliferative capacity and a resistance to undergo apoptosis (desJardins‐Park et al., 2018).

Formation of ECM during MI wound healing is very similar to skin wound healing. Fibroblasts follow a similar trajectory of recruitment to the injury site, polarization to reparative fibroblasts, proliferation, and synthesis and deposition of ECM (Liehn et al., 2011). Similar to skin wound healing, the provisional matrix is replaced initially with ECM rich in collagen type III, which is replaced later with collagen type I and crosslinked to strengthen the scar (Czubryt, 2012; Daseke et al., 2020) During MI wound healing, insufficient ECM synthesis and deposition can result in a weak scar and cardiac rupture, whereas excessive ECM deposition can cause arrythmias, a stiff ventricle and diastolic dysfunction (Chalise et al., 2023).

### 6.2 Angiogenesis

Angiogenesis is necessary for oxygen and nutrient supply to tissues and is thus a crucial part of the healing process after both a skin injury and MI (Rodero & Khosrotehrani, 2010). Angiogenesis starts between 2 and 4 days after injury and occurs mainly thorough sprouting of pre‐existing vessels (Tonnesen et al., 2000; Wu et al., 2021). Two factors known to stimulate angiogenesis after both a skin injury and MI are VEGFA and FGF2 (Rodero & Khosrotehrani, 2010; Tonnesen et al., 2000; Wu et al., 2021). VEGFA is a highly potent angiogenic factor released by macrophages, fibroblasts, keratinocytes and cardiomyocytes (Martin, 1997; Tonnesen et al., 2000; Wu et al., 2021). VEGFA recruits endothelial cells to the wounded area and stimulates proliferation to promote angiogenesis. FGF2 is released by damaged endothelial cells and by macrophages, and induces angiogenesis by stimulating production of hypoxia‐inducible factor 1‐alpha and VEGFA (Martin, 1997; Seghezzi et al., 1998). An important step in revascularization is to turn off the stimulation for angiogenesis, to prevent excessive vessel formation. Thrombospondin is a crucial factor, because the day 7 MI fibroblast secretome represses angiogenesis through thrombospondin 1 signalling (Mouton et al., 2019).

### 6.3 Re‐epithelialization

Re‐epithelialization starts to occur within hours after injury, with signalling already occurring to stimulate the process. Keratinocytes migrate from the wound edges and under the blood clot to bridge the gap (Rodero & Khosrotehrani, 2010). Upon injury, immune cells infiltrating the wound, in addition to fibroblasts and other cells at the injury site, release cytokines and growth factors that activate keratinocytes. Activation of keratinocytes stimulates proliferation and migration of the cells from the wound edge and over the denuded area (Pastar et al., 2014). Epidermal growth factor (EGF), TGF‐α and heparin‐binding epidermal growth factor directly stimulate keratinocyte proliferation and migration through the EGF receptor (Pastar et al., 2014). FGF is also known as keratinocyte growth factor (KGF) and stimulates keratinocyte migration and proliferation by binding to the KGFR2IIIb receptor found exclusively on keratinocytes (Martin, 1997; Pastar et al., 2014). KGF is produced by fibroblasts and increases >100‐fold within 24 h of injury (Martin, 1997; Pastar et al., 2014). Pro‐inflammatory cytokines, such as IL‐1β, IL‐6 and TNF‐α, can indirectly modulate keratinocyte migration and proliferation by regulating fibroblast secretion of growth factors, such as KGF (Pastar et al., 2014). Impaired re‐epithelialization is seen in all types of chronic skin wounds (Pastar et al., 2014). This process is where skin wound healing diverges from cardiac wound healing, with little to no regeneration occurring in the case of the cardiac response to MI.

## 7 CONCLUSION

The similarities in the wound‐healing response between the skin and the heart allow us to use the response to skin wounding to inform how one would respond to MI. Those with faster skin wound healing have better wound healing and would be more likely to survive MI and to repair more effectively. Of course, clinically, there remains much work to be done before an algorithm or risk score calculation could be derived. Co‐morbidities need to be taken into account, particularly diabetes, which is well known to cause wound‐healing deficits (Baltzis et al., 2014). For patients with MI, timely reperfusion is the current best therapy, and failure to restore blood flow in a timely manner can result in poor cardiac wound healing with adverse remodelling, yielding heart failure over time. Thus, how timely the response is has critical consequences. Being able to capitalize on commonalties among inflammation, resolution, angiogenesis and ECM‐formation processes regulated in a similar manner across tissues has implications for MI therapy, in addition to organ damage in other systems, such as renal and neural pathologies. The ability of wound healing to be compared across organs provides us with a prognostic tool, in addition to a more amenable model in which to test the response to therapies as first‐pass evaluations.

## AUTHOR CONTRIBUTIONS

Merry L. Lindsey: Conceptualization (lead); project administration (lead); funding acquisition (lead); writing—original draft (lead); and writing—review and editing (lead). Mediha Becirovic Agic: Conceptualization (lead); project administration (lead); funding acquisition (supporting); writing—original draft (lead); and writing—review and editing (lead). Both authors approved the final version of the manuscript and agree to be accountable for all aspects of the work in ensuring that questions related to the accuracy or integrity of any part of the work are appropriately investigated and resolved. Both persons designated as authors qualify for authorship, and all those who qualify for authorship are listed.

## CONFLICT OF INTEREST

The content is solely the responsibility of the authors and does not necessarily represent the official views of any of the funding agencies.
